# An alternative explanation to the unusual neon pink color in a radiotherapy tattoo

**DOI:** 10.1016/j.jdcr.2026.07.070

**Published:** 2026-08-23

**Authors:** Nicolas Kluger

**Affiliations:** a Department of Dermatology, “Tattoo” Consultation, Bichat-Claude Bernard Hospital, Assistance Publique-Hôpitaux de Paris, Paris, France; b Dermatology, Aava Medical Center, Kerava, Finland; c Department of Dermatology, Tampere University Hospital, Tampere, Finland

**Keywords:** radiotherapy, tattooing, ultraviolet tattoo

*To the Editor:* I read with interest the article by Boroumand *et al*, describing a 66-year-old woman presenting with autofluorescent neon pink pigmentation at the site of radiotherapy tattoos placed 3 years earlier.^1^ The authors proposed that “chemical modification and interactions between ink components may have occurred in unexpected ways, altering the tattoo's appearance.”^1^ However, black tattoo inks (CI 77266, Indian ink) consist solely of carbon black plus additives^2^; they are not, as the authors suggest, a blend of multiple-colored pigments. No known chemical process can convert carbon black into a pink, UV-reactive pigment. Since tattoos persist for a lifetime or fade only very gradually, it is striking that after just 3 years no black pigment whatsoever remained visible in the skin. The hypothesis of a dark pigment that would have progressively and completely faded away within 3 years without the patient’s knowledge is highly unlikely. Furthermore, one would expect to find some remaining pigments in the dermis. The use of luminescent ink for radiotherapy marking is a documented practice.^3^^,^^4^ The simplest and most plausible explanation is that the neon pink pigment was introduced into the skin through tattooing itself. Notably, InkAlign markets in North America a single-use medical tattooing device specifically using pink UV-reactive ink for radiotherapy marking.^5^ Rather than an unexplained chemical transformation of standard black ink, the authors should contact the radiotherapy center where the patient was treated to clarify their marking protocol and confirm which ink product was used.

### Declaration of generative AI and AI-assisted technologies in the writing process

Claude (Anthropic) was used to assist with language editing of this manuscript. The author is fully responsible for the content and conclusions presented.

## Conflicts of interest

None disclosed.
